# Social marginalisation, environmental degradation and *Toxoplasma gondii* exposure in urban informal settlements in Brazil

**DOI:** 10.1371/journal.pntd.0014453

**Published:** 2026-06-22

**Authors:** Max T. Eyre, Joyce Y. Wang, Ianei de O. Carneiro, Renato B. Reis, Elsio A. Wunder, Nivison N. Júnior, Guilherme S. Ribeiro, Fabio N. Souza, Juliet O. Santana, Ellie A. Delight, Ridalva D. M. Felzemburgh, Francisco S. Santana, Sharif Mohr, Astrid X. T. O. Melendez, Adriano Queiroz, Andreia C. Santos, Jaqueline S. Cruz, Meghan Owens, Bruno Martorelli Di Genova, Mitermayer G. Reis, Claudia Muñoz-Zanzi, Peter J. Diggle, Federico Costa, Albert I. Ko

**Affiliations:** 1 Environmental Health Group, London School of Hygiene & Tropical Medicine, London, United Kingdom; 2 Yale School of Public Health, New Haven, Connecticut, United States of America; 3 Institute Gonçalo Moniz, Oswaldo Cruz Foundation, Ministry of Health, Salvador, Brazil; 4 School of Veterinary Medicine and Animal Science, Federal University of Bahia, Salvador, Brazil; 5 Department of Pathobiology and Veterinary Science, University of Connecticut, Storrs, Connecticut, United States of America; 6 Faculty of Medicine, Federal University of Bahia, Salvador, Brazil; 7 Institute of Collective Health, Federal University of Bahia, Salvador, Brazil; 8 State Secretary of Health of Bahia, Salvador, Brazil; 9 Department of Microbiology and Molecular Genetics, University of Vermont, Burlington, Vermont, United States of America; 10 Division of Environmental Health Sciences, School of Public Health, University of Minnesota, Minneapolis, Minnesota, United States of America; 11 Centre for Health Informatics, Computing, and Statistics, Lancaster University Medical School, Lancaster, United Kingdom; NHS Blood and Transplant, UNITED KINGDOM OF GREAT BRITAIN AND NORTHERN IRELAND

## Abstract

*Toxoplasma gondii* infection poses a substantial global health burden, yet transmission pathways and population susceptibility in urban informal settlements remain poorly characterised, particularly for women of childbearing age. We analysed archived samples from a cross-sectional serosurvey of 728 children and adolescents aged 4–18 years living in a marginalised urban community in Salvador, Brazil, to characterise exposure patterns and identify demographic, socioeconomic, behavioural, household, and environmental factors associated with seropositivity and to assess spatial heterogeneity in exposure risk. Overall seroprevalence was 49%, increasing with age and higher in males than females; Bayesian serocatalytic models estimated sex-specific forces of infection of 0.078 for males and 0.050 for females, with approximately half of female participants still susceptible upon reaching childbearing age, highlighting the risk of congenital toxoplasmosis. In regression analyses, seropositivity was associated with male sex, lower household income, cat ownership, and residence at lower elevation, greater distance from the main road, and reported contact with sewer water. Notably, most seropositive participants (77.3%) did not live in households with cats. Geostatistical modelling demonstrated fine-scale spatial heterogeneity, with clustered hotspots exceeding 50–60% predicted prevalence. Adjustment for measured covariates attenuated but did not eliminate spatial clustering, indicating residual fine-scale spatial structure consistent with unmeasured environmental processes operating beyond individual households, alongside additional unstructured variation that may reflect household-level or peridomestic differences not captured by the measured covariates. Together, these findings provide evidence consistent with an important role for household and peridomestic environmental exposure pathways in *T. gondii* transmission in informal settlements, extending beyond households with domestic cats and shaped by social marginalisation and environmental vulnerability.

## Author summary

*Toxoplasma gondii* is a parasite that infects a large proportion of the global population and can cause severe disease when infection occurs during pregnancy. However, little is known about how people are exposed to *T. gondii* in urban informal settlements, where living conditions may increase contact with environmental sources of infection. We analysed blood samples collected from children and adolescents living in a low-income urban community in Salvador, Brazil, to understand patterns of exposure and identify factors associated with infection. Nearly half of participants showed evidence of past infection, and approximately half of girls remained uninfected upon reaching childbearing age, highlighting ongoing risk of congenital toxoplasmosis. Infection was more common among boys, children from lower-income households, and those reporting contact with sewer water or living in lower-lying areas further from main roads. Notably, most infected children did not live in households with cats, suggesting that infection risk was not confined to homes with domestic cats. Using spatial analyses, we identified clusters of high exposure risk that persisted even after accounting for measured risk factors, indicating that unmeasured environmental processes contribute to exposure beyond individual households. At the same time, additional variation occurred at very small spatial scales, consistent with the influence of household and peridomestic conditions. Together, our findings suggest that exposure to *T. gondii* in informal urban settlements is shaped by social marginalisation and environmental vulnerability, with contamination within and around households likely driving risk. These findings add to limited evidence on *T. gondii* exposure pathways in urban informal settlements and may inform efforts to reduce infection risk in similar communities.

## Introduction

*Toxoplasma gondii* is an obligate intracellular protozoan with a worldwide distribution [1] and an estimated global average prevalence of 25% [2]. It is an important One Health problem with a substantial global public health burden that is disproportionately concentrated in low-income urban and rural populations [3,4]. A large proportion of this burden arises from congenital toxoplasmosis, for which the global incidence is estimated to be 190,100 cases per year (1.5 per 1000 live births), corresponding to approximately 1.20 million disability-adjusted life-years (DALYs) per year [3].

Human infection occurs via oral or congenital routes. Exposure can result from ingestion of environmentally resistant oocysts contaminating soil, food, or water, consumption of tissue cysts (bradyzoites) in raw or undercooked meat from infected animals, or vertical transmission during pregnancy [5]. Oocysts are shed in the faeces of infected felines - the definitive hosts of *T. gondii* - and can persist in the environment for months to over a year, remaining resistant to freezing, drying, and disinfectants [6,7]. As a result, environmental contamination can constitute a sustained source of infection for humans and animals, particularly in mixed-environment settings with limited hygiene infrastructure and close human–animal contact.

The public health importance of toxoplasmosis is driven primarily by its impact on pregnant women, their offspring, and immunocompromised individuals. While infection in immunocompetent hosts is often asymptomatic or mild [8], primary infection during pregnancy can result in congenital toxoplasmosis, leading to miscarriage, hydrocephalus, central nervous system abnormalities, and chorioretinitis [9,10]. Disease severity is inversely related to gestational age at infection, with first- and second-trimester infections more likely to cause severe outcomes [9,10]. Ocular toxoplasmosis, a leading cause of chorioretinitis globally, may present years after either congenital or postnatal infection [9,11]. Women of childbearing age are therefore a critical population for prevention efforts. In immunocompromised individuals, toxoplasmosis may cause life-threatening complications such as encephalitis and pneumonitis [12]. Emerging evidence also suggests potential links between latent infection and neuropsychiatric disorders, indicating a broader disease burden than previously recognised [13].

Brazil has one of the highest burdens of *T. gondii* infection globally. Seroprevalence estimates suggest exposure in up to 50% of school-aged children and 50–80% of women of childbearing age, with congenital infection rates estimated at 5–23 per 10,000 births [14]. Brazil has also experienced a disproportionately high number of documented toxoplasmosis outbreaks over the past five decades [15]. This elevated transmission is thought to reflect a convergence of socio-environmental factors common in low-income populations, including contaminated food and water, inadequate sanitation, poor hygiene, free-roaming domestic cats, and the circulation of atypical and more virulent *T. gondii* genotypes [4,15,16]. These genotypes, which are uncommon in Europe, have been associated with more severe clinical outcomes among congenitally infected children in Brazil [4,17].

Urban informal settlements in Brazil, and globally, are likely to be settings of heightened vulnerability to *T. gondii* infection [18–22], with women of childbearing age in these communities at particular risk. Rapid and uneven urbanisation in Brazil has led to the expansion of informal settlements over recent decades, a trend mirrored worldwide with the global population living in informal settlements projected to reach three billion by 2030 [23]. Despite the size and vulnerability of this population, the epidemiology of *T. gondii* in informal urban settings remains poorly characterised. Previous studies have found that socioeconomic vulnerability is associated with exposure [19], but little is known about the mechanisms by which social marginalisation drives risk within informal settlements.

There are consequently two important research priorities for *T. gondii* in marginalised urban populations [4,17]. First, robust estimates of seroprevalence in children and women reaching reproductive age, together with age- and sex-specific transmission rates, are needed to identify populations at risk of primary infection during pregnancy and to understand how transmission dynamics vary across demographic groups and settings. Second, detailed characterisation of exposure risk is required to clarify the social and environmental pathways through which transmission occurs, including whether infection is primarily associated with households that own cats, or instead reflects shared peridomestic and environmental exposures extending beyond individual households, or food-borne exposure through contaminated meat. Characterising transmission at the human–animal–environment interface is therefore critical for informing effective, community-based prevention strategies.

In this study, we analysed archived samples from a cross-sectional serosurvey conducted in 2003 among children and adolescents aged 4–18 years living in an urban low-income community in Brazil. These data provide a valuable opportunity to address the research priorities outlined above, combining serological outcomes with detailed demographic, behavioural, and environmental information for a highly exposure-informative age group. Given the paucity in available evidence, historical datasets from these settings are valuable for establishing transmission benchmarks, contextualising contemporary findings, and identifying exposure pathways that may remain relevant in present-day informal settlements with similar social and environmental conditions. The aims of this study were: 1) describe trends in *T. gondii* seroprevalence within the study population and estimate sex-specific force of infection; 2) identify demographic, socioeconomic, behavioural, household, and environmental factors associated with seropositivity; and 3) characterise fine-scale spatial heterogeneity in exposure risk and assess the relative contributions of household-level and environmental processes.

## Methods

### Ethics statement

Participants were enrolled according to written informed consent. For participants under 18 years of age, written informed consent was obtained from a parent or legal guardian. The collection and testing of samples for *T. gondii* was approved by the Ethics Committee of the Centro de Pesquisas Gonçalo Moniz, Fundação Oswaldo Cruz (CEP-CPqGM/FIOCRUZ), under the authority of the Brazilian National Research Ethics Commission (CONEP), in accordance with Resolution 196/96 of the National Health Council (protocols no. 89, approval no. 06/2002).

### Study site and population

This study was conducted in Pau da Lima, a marginalised urban community located on the periphery of the city of Salvador, Northeast Brazil. The study site consisted of four distinct valleys with a total area of 0.46 km^2^, which are characterised by large elevation gradients, high population density, inadequate sanitation and drainage infrastructure, and a mixed peri-urban environment of soil, vegetation and paved surfaces.

The study population was initially recruited for a seroprevalence study of leptospirosis in 2003, with results published previously [24]. In this study we re-analysed previously collected serum samples for a subset of participants aged 4–18 years to measure seroprevalence for *T. gondii*-specific antibodies. The study population was recruited as follows (see S1 Fig). In 2003, a preliminary census was conducted for the study area, identifying 14,122 inhabitants living in 3,689 households, of which 12,651 were eligible to participate in the study. The eligibility criteria were: residing for at least three nights in the previous week in a household within the study area; being aged four years or older; and providing written informed consent for participation in the study. Households containing eligible subjects were assigned sequential numbers, and a computer-based random generator was used to select a subset of 861 households consisting of 2,003 eligible residents who were invited to participate in the study between April 2003 and May 2004. From these households, 749 participants aged 4–18 years were successfully recruited. The sample size was further reduced to 728 subjects because serum samples were not available for all participants.

### Data collection

#### Household survey.

A team of community healthcare workers, nurses, and physicians administered a standardised questionnaire during home visits to collect participant data on demographic and socioeconomic status (SES) indicators and self-reported environmental exposures. Participants self-reported their race. Household income and ownership were determined through interviews with the household head. The study team surveyed the household area to determine the presence of dogs, cats, and chickens, and vegetation in the peridomestic area. Household locations were georeferenced during the original field survey using handheld GPS units.

#### Mapped environmental variables.

To create additional environmental variables, household coordinates were collected during study site house visits and the study team mapped sites with large trash dumps, the open sewer network and main roads. The shortest distance from each household to each of these three environmental features was calculated for use as an explanatory variable. The variable ‘distance to main road’ is a known proxy for social and environmental marginalisation in these communities due to the concentration of poverty, limited infrastructure, and reduced access to public services in areas further from main roads [24]. Household elevation was also extracted for each household from a 5m resolution digital elevation model provided by the Salvador municipal government, with lower elevation in the valleys a proxy for flooding risk, a more mixed and less paved environment (including exposed soil), which are characteristic of environmentally degraded areas and may increase environmental exposure [25].

#### Serological analysis.

Serum samples were obtained from the blood collected from the subjects during home visits. The *T. gondii* IgG enzyme immunoassay (EIA) kit (BioRad, Hercules, CA) was used to evaluate serological evidence of prior infection. Samples that produced equivocal results were retested to determine whether a seropositive or seronegative outcome was obtained. If the sample remained undetermined, it was classified as seronegative.

#### Grouping of explanatory variables by domain.

Explanatory variables were grouped into four domains - demographic and socioeconomic characteristics, household animals, household and peridomestic environment, and contact with the environment - to reflect both potential sources of *T. gondii* oocysts and the settings in which exposure may occur (see S1 Table for full definitions and rationale). This framework was used to assess social and environmental drivers of exposure, and whether exposure was more consistent with household-based pathways or with environmental and peridomestic exposure pathways operating beyond individual households.

### Statistical analysis

#### Age- and sex-specific seroprevalence and force of infection.

To characterise exposure to *T. gondii* across childhood, we estimated age- and sex-specific seroprevalence by calculating the proportion of participants with *T. gondii* IgG antibodies across five age groups (4–6, 7–9, 10–12, 13–15, and 16–18 years), stratified by sex. Confidence intervals were calculated using exact binomial methods.

We then fitted a serocatalytic model [26] to estimate the annual force of infection (FOI), defined as the per-capita rate at which susceptible individuals become infected, and the corresponding annual infection probability (AIP), representing the proportion of susceptible individuals expected to be infected each year, in male and female participants. The catalytic model assumes that susceptible individuals are infected at a constant FOI, λ, across their lifetime (i.e., independent of age (a) and calendar year) and that once they have been infected, they recover and remain immune and seropositive. The proportion seropositive at age a, z(a), is given by


$$\begin{array}{c} {z(a)=\text{1}-e}^{-\lambda a} \end{array}$$

The corresponding mean AIP is then calculated as follows:


$$\begin{array}{c} {AIP=\text{1}-e}^{-\lambda } \end{array}$$

To estimate sex-specific FOI and AIP, we used Bayesian inference to fit this serocatalytic model to empirical data on male and female participants separately following the methodology previously described by Rees et al. [27]. A uniform distribution between 0 and 0.5 was used as an uninformative prior for FOI. Model parameters were estimated using Markov chain Monte Carlo (MCMC) with the Gibbs sampling algorithm, implemented in RJags (version 4–10) [28]. We used the Gelman-Rubin statistic to evaluate MCMC convergence considering a threshold of <1.1 [29]. The predicted relationship between seroprevalence and age and 95% credible intervals (CrI) was plotted for each sex.

As a sensitivity analysis we assessed whether exposure was age-dependent and changed after reaching adolescence for female participants by fitting a piecewise serocatalytic model that allowed the force of infection to differ before and after age 12.

#### Factors associated with seropositivity.

We calculated descriptive statistics and seroprevalence for variables across the four domains (demographic and socioeconomic characteristics, household animals, household and peridomestic environment, and contact with the environment). Univariable logistic mixed-effects regression models with a random intercept at the household level were used to estimate the crude associations between each variable and the odds of *T. gondii* seropositivity. All continuous explanatory variables were initially assessed for non-linearity with the log-odds of seropositivity using generalized additive models (GAMs) [30]; where no strong departures from linearity were observed, variables were modelled linearly (S2 Fig).

**Fig 1:**
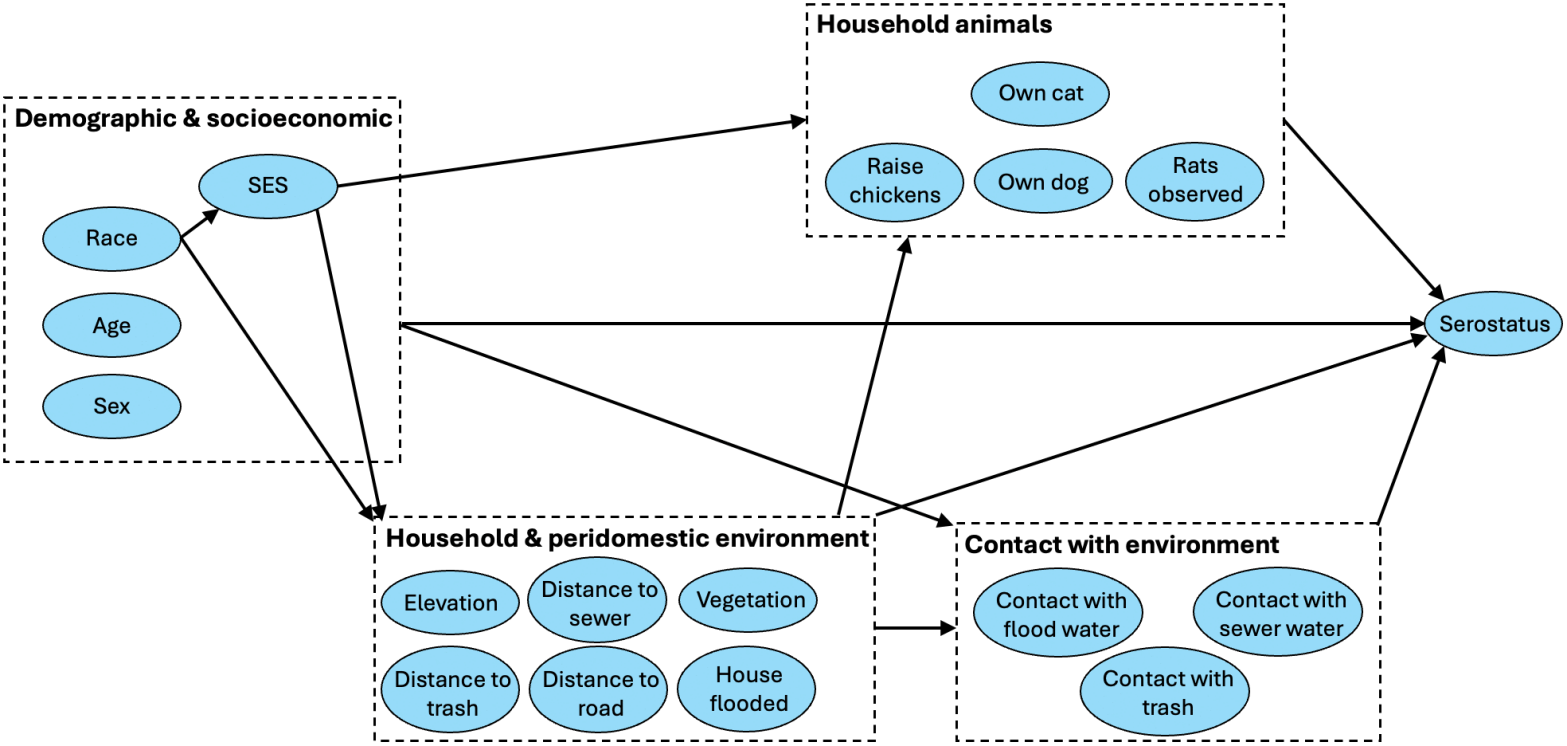
Simplified Directed Acyclic Graph (DAG) for *T. gondii* serostatus in children and adolescents. Variables are shown grouped by their domains with arrows between domain boxes representing the relationship between all variables within those domains. Within domain relationships are not shown except for race and socioeconomic status (SES). A single SES indicator, per-capita household income, was used for this analysis.

Multivariable regression analyses were guided by a causal framework to examine the relationship between exposures within each domain and *T. gondii* seropositivity. Assumed relationships between variables were formalised using a Directed Acyclic Graph (DAG) constructed in Dagitty [31]; a simplified version is shown in Fig 1, with the full DAG provided in S3 Fig and available online (https://dagitty.net/m5z6UTbAP). All causal language used herein follows the principles outlined by Tennant et al. [32]. We assumed that demographic and socioeconomic characteristics (age, sex, race, household income, and house ownership) may influence serostatus both directly and indirectly. Race and socioeconomic characteristics were treated as upstream structural determinants that shape where a child lives and, in turn, influence animal ownership and conditions in the household and peridomestic environment, as well as opportunities for contact with the environment near the household. In contrast, child age and sex were not assumed to influence environmental conditions but may affect exposure through behavioural pathways captured within the contact-with-environment domain. Conditions in the household and peridomestic environment were assumed to influence children’s likelihood of contact with potentially contaminated environments near the home. Finally, the household animals, household and peridomestic environment, and contact with the environment domains were assumed to affect serostatus directly through increased opportunities for exposure to *T. gondii* oocysts.

Multivariable logistic regression models with a random intercept at the household level were used to estimate the total effect of each exposure on seropositivity, as defined by the assumed causal structure in the DAG, adjusting for the sufficient adjustment sets identified in the DAG [31,32]. Collinearity among variables was assessed using Variance Inflation Factor (VIF). Variables with VIF values exceeding 5 were considered to exhibit potentially problematic multicollinearity, but this was not exceeded for any variables. While our cross-sectional design precludes causal claims, the use of DAGs enables transparent specification of the estimand and principled identification of adjustment sets to minimise confounding. Our estimates should be interpreted as associations corresponding to the total effect under the assumed causal structure, contingent on the assumptions specified in the DAG.

E-values were calculated for estimated odds ratios to assess the minimum strength of association that an unmeasured confounder would need to have with both the exposure and seropositivity, conditional on measured covariates, to fully explain away the observed associations [33]. To aid interpretation of odds ratios, we estimated model-based marginal predicted seroprevalence for selected key binary exposures by averaging predicted probabilities from the fitted mixed-effects models over the observed distribution of covariates.

#### Spatial modelling of heterogeneity in seroprevalence.

We examined spatial heterogeneity in *T. gondii* seroprevalence using binomial geostatistical models implemented in the PrevMap package in R [34]. Full details of the geostatistical modelling framework, including the model specification, likelihood estimation by Monte Carlo maximum likelihood, and the prediction procedure, are provided in S1 Appendix.

Individual serostatus was modelled as a Bernoulli outcome with a logit link (Eq. (1)), where $p_{j}\left(x_{i}\right)$ denotes the probability that individual j residing at household location $x_{i}$ was seropositive. The linear predictor included fixed effects for measured covariates, comprising location-specific covariates $d(x_{i})$ with regression coefficients β, and individual-level covariates $e_{ij}$ with regression coefficients γ a spatially correlated Gaussian process S(x), and an unstructured random effect $Z_{i}$. The spatial process S(x) was assumed to have mean zero and variance $\sigma^{\text{2}}$, capturing residual spatial variation in seroprevalence not explained by measured covariates, with spatial correlation governed by a range parameter ϕ that determines the rate at which correlation decays with distance. The term $Z_{i}$ was assumed to have variance $\tau^{\text{2}}$ and captures unstructured variation arising from a combination of spatial processes operating at scales below the sampling resolution and within-household extra-binomial variability, collectively represented by the nugget effect.


$$\begin{array}{c} ogit(p_{j}\left(x_{i}\right))=d{\left(x_{i}\right)}^{⊤}\beta +\;{e_{ij}}^{⊤}\gamma +S\left(x_{i}\right)+Z_{i} \end{array}$$ (1)

First, an intercept-only geostatistical model was fitted to characterise the overall spatial distribution of seroprevalence. Seroprevalence was predicted on a 4 m × 4 m grid across the study area. From posterior samples of predicted prevalence, we derived (i) the posterior mean predicted prevalence at each location and (ii) the posterior probability that prevalence exceeded 50% (exceedance probability).

We then fitted a full geostatistical model including covariates to examine residual spatial variability not captured by measured risk factors. Variable selection for this prediction model proceeded as follows: only variables associated at the 5% significance level in the multivariable regression analysis were considered. Logistic mixed-effects regression models were fitted for all possible combinations of these variables, and the model with the lowest Akaike Information Criteria (AIC) [35] value was selected. The selected variables were included in the geostatistical model, and the posterior mean of the residual spatial process S(x) was predicted across the study area to visualise spatial clustering in risk not explained by measured covariates. The proportion of residual variation that was spatially structured was quantified as $\sigma^{\text{2}}/(\sigma^{\text{2}}+\tau^{\text{2}})\;$where $\sigma^{\text{2}}$ represents the variance of the spatially structured Gaussian process and $\tau^{\text{2}}$ represents unstructured variation arising from processes operating at spatial scales finer than the sampling resolution, including within-household or immediate peridomestic heterogeneity not captured by the spatial process.

### Data and code availability

All data and R code used to conduct this analysis are available at https://github.com/maxeyre/Toxo-PdL-children. To enable public data sharing while preserving anonymity, age was grouped into three-year bands, household coordinates were removed, and variables with sparse categories were merged.

## Results

### Description of the study population

The study population (Table 1) was 49.3% male with an even distribution of participants across age groups: 4–6 years (15.2%), 7–9 years (22.3%), 10–12 years (19.6%), 13–15 years (19.2%) and 16–18 years (23.6%). Most participants self-identified as Pardo (mixed race; 62.5%), followed by Black (32.4%) and White (4.8%). A majority (87.6%) of participants lived in households for which their family did not own the title to their home. The median daily household per-capita income was US$0.61. Household elevation varied considerably, between 25m and 79m, a result of the steep inclines within the study areas.

**Table 1:** Study population characteristics, seroprevalence and univariable associations between variables and *T. gondii* seropositivity.

	Total (n = 728)	Seroprevalence	Univariable
	No. of individuals (%) or median (IQR)^1^	No. of seropositive individuals (%) or median (IQR) among seropositive^1^	OR (95% CI)
**Demographic & socioeconomic**			
Age (continuous)	11 (8,15)	13 (9,16)	1.22 (1.15, 1.29)
Age (years)			
4-6	111 (15.2%)	26 (23.4%)	REF
7-9	162 (22.3%)	66 (40.7%)	2.87 (1.43, 5.76)
10-12	143 (19.6%)	64 (44.8%)	4.22 (2.01, 8.85)
13-15	140 (19.2%)	91 (65.0%)	11.77 (5.31, 26.09)
16-18	172 (23.6%)	110 (63.9%)	11.35 (5.23, 24.65)
Sex			
Female	369 (50.7%)	157 (42.5%)	REF
Male	359 (49.3%)	200 (55.7%)	2.05 (1.4, 3.02)
Race			
Pardo (mixed)	455 (62.5%)	206 (45.3%)	REF
Black	236 (32.4%)	141 (59.7%)	1.93 (1.27, 2.94)
White	35 (4.8%)	9 (25.7%)	0.28 (0.10, 0.77)
Other	2 (0.3%)	1 (50.0%)	2.21 (0.07, 74.58)
Per capita daily household income (US$)	0.61 (0.17, 1.05)	0.51 (0.17, 0.96)	0.67 (0.49, 0.92)
House is rented	27 (3.7%)	13 (48.1%)	1.09 (0.39, 3.04)
Owns the title to the household	90 (12.4%)	42 (46.7%)	0.81 (0.42, 1.54)
**Household animals**			
Cat in household	133 (18.3%)	81 (60.9%)	2.00 (1.16, 3.43)
Dog in household	325 (44.6%)	171 (52.6%)	1.29 (0.85, 1.97)
Raise chickens	290 (39.8%)	164 (65.5%)	1.68 (1.12, 2.53)
Observed rats in or near house	562 (77.2%)	298 (53.0%)	2.05 (1.31, 3.20)
**Household & peridomestic environment**			
Household elevation (m)	50.4 (39.1, 62.2)	46.1 (37.0, 59.7)	0.70 (0.59, 0.82)^2^
Distance to the main road (m)	164.4 (94.2, 282.8)	200.4 (107.7, 323.2)	1.23 (1.13, 1.35) ^3^
House flooded in last 6 months	95 (13.0%)	57 (60.0%)	1.77 (0.95, 3.32)
Distance to nearest trash dump (m)	78.9 (51.2, 115.7)	75.1 (48.1, 112.8)	0.96 (0.91, 1.01) ^2^
Distance to nearest open sewer (m)	19.7 (7.5, 38.8)	15.0 (7.1, 32.4)	0.82 (0.74, 0.91) ^2^
Vegetation within 10m of the house	513 (70.5%)	261 (50.9%)	1.45 (0.90, 2.33)
**Contact with environment**			
Contact with sewer water	202 (27.7%)	127 (62.9%)	2.63 (1.66, 4.18)
Contact with trash	172 (23.6%)	97 (56.4%)	1.46 (0.92, 2.31)
Contact with flood water	344 (47.3%)	183 (53.23%)	1.34 (0.91, 1.98)

^1^Numbers and percentages are shown for categorical variables. Median and interquartile range (IQR) are shown for continuous variables; ^2^OR shown for an increase in 10m; ^3^OR shown for an increase in 50m.

A total of 133 (18.3%) participants lived in households that owned cats. The majority of participants (n = 562, 77.2%) had observed rats near to their household. Contact with potential environmental sources of exposure was common, with 47.3% and 27.7% of participants reporting recent contact with flood water and sewer water, respectively, near the house. In this setting, contact with sewer water occurs through exposure to the open sewer channels running through the community, which residents may encounter when moving around the neighbourhood.

### Age- and sex-specific seroprevalence and force of infection

**Fig 2:**
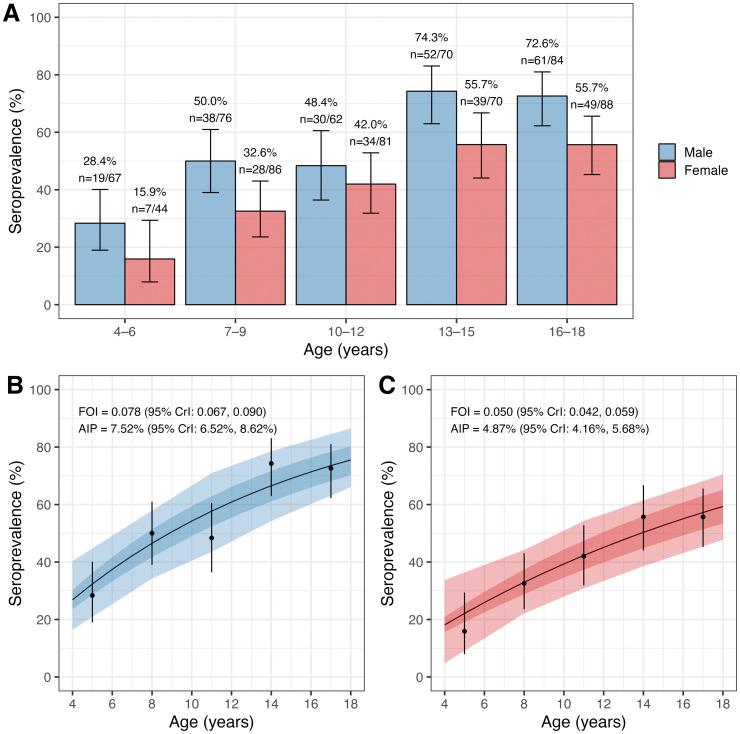
Age- and sex-specific *T. gondii* seroprevalence and serocatalytic model estimates. **A)** Distribution of seroprevalence by age group and sex. Error bars represent 95% confidence intervals; **B-C)** Serocatalytic model estimates of age-specific seroprevalence for **B)** male and **C)** female participants with 95% credible intervals (dark shading) and binomial sampling uncertainty (light shading). Estimated annual force of infection (FOI) and corresponding annual infection probability (AIP) are shown on each panel. The observed proportion of seropositive individuals is plotted for each 3-year age group (black points) with error bars representing 95% confidence intervals.

Among 728 study participants, we identified serological evidence of previous exposure to *T. gondii* in 357 individuals and a crude seroprevalence of 49.0%. Seroprevalence increased with age, from 23.4% in individuals aged 4–6 years to 63.9% in the oldest group aged 16–18 years (Table 1). Seroprevalence was higher in male than in female participants (55.7% and 42.5%, respectively), and this sex disparity was observed in all age groups (Fig 2). For both males and females, seroprevalence was similar in the 13–15 and 16–18 age groups.

Sex-specific serocatalytic models estimated the annual force of infection (FOI) to be 0.078 (95% credible interval (CrI) 0.067, 0.090) and 0.050 (95% CrI 0.042, 0.059) for male and female participants, respectively. These FOIs were equivalent to annual infection probabilities (AIPs) of 7.52% (95% CrI 6.52%, 8.62%) in male participants and 4.87% (95% CrI 4.16%, 5.68%) in female participants.

Model predictions are shown for males and females in Fig 2B and 2C, respectively, and suggest that approximately 40% of female participants remained unexposed by age 18 years. The sensitivity analysis using a piecewise FOI model supported the constant-FOI assumption, with no evidence of reduced exposure after age 12. In this model, the estimated FOI was 0.049 before age 12 (95% CrI 0.039, 0.060) and 0.053 after age 12 (95% CrI 0.008, 0.109), with a posterior probability of 0.46 that the post-12 FOI was lower than the pre-12 FOI. Together, these findings suggest that many girls remain susceptible as they enter reproductive age while continuing to live in a high-transmission environment, with potential implications for the risk of primary infection during pregnancy.

### Factors associated with seropositivity

**Fig 3:**
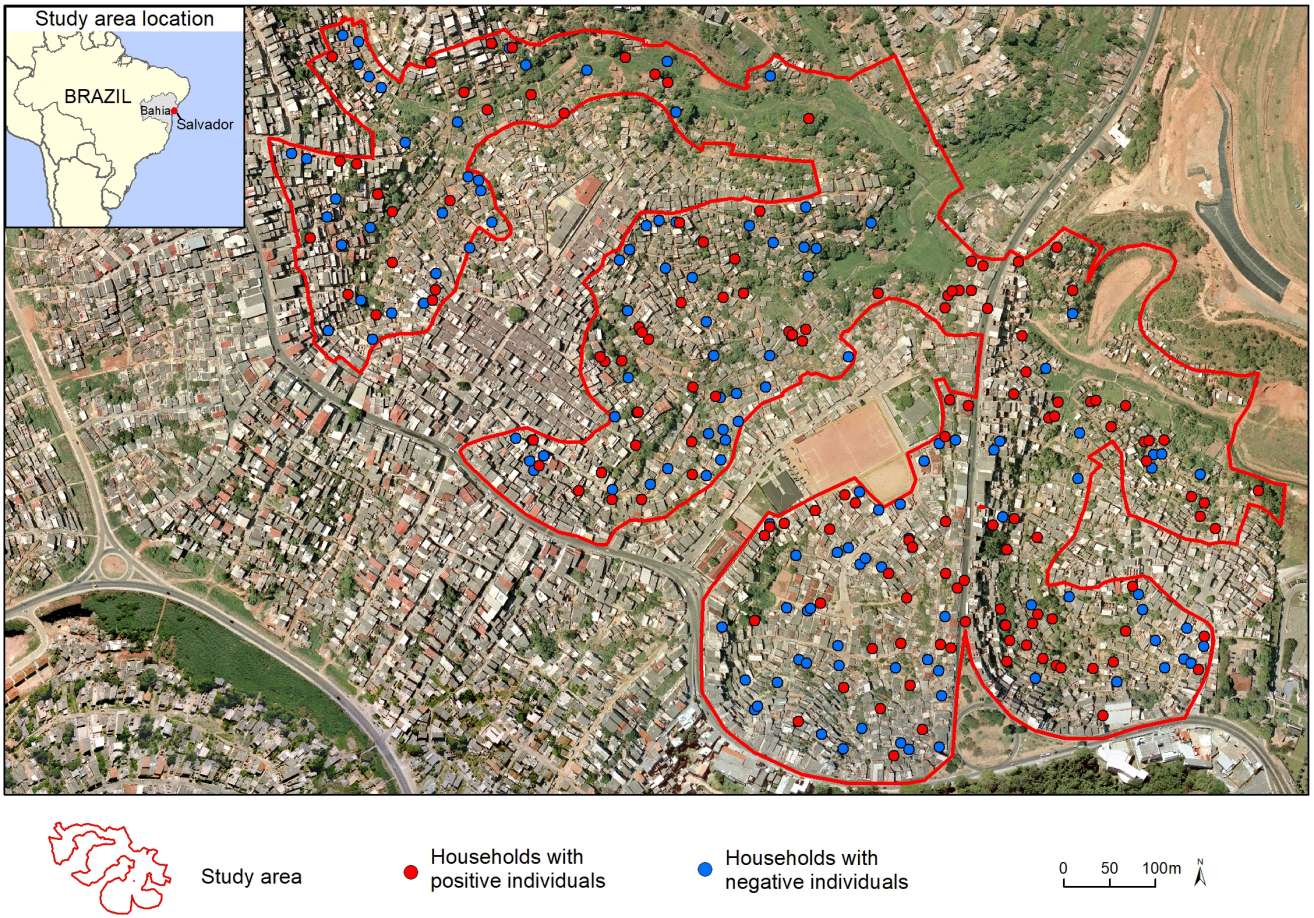
Approximate locations of participant households within the study area, with *T. gondii* serostatus indicated by circle colour (blue – negative; red – positive). Locations were randomly displaced to preserve anonymity. The inset shows the location of Salvador in Northeast Brazil. Source: Salvador/CONDER cartographic base map obtained from Geopolis, Infraestrutura de Dados Espaciais da Bahia (IDE Bahia), Governo do Estado da Bahia using CONDER/INFORMS open cartographic data available under the Open Data Commons Attribution License (ODC-By) [36]; state limits for inset image from publicly available IBGE Malhas Territoriais, 2017 [37].

Seropositivity appeared to cluster within some households, with 22.8% of households having two or more seropositive children, while 42.1% had none and 35.1% had one. Mean household seroprevalence, calculated as the number of seropositive participants in the household divided by the total number of participants in the household, was 44.8% across all study households and 77.5% in study households with at least one seropositive individual. Fig 3 shows the distribution of *T. gondii* serostatus across the three valleys.

Higher seroprevalence was observed among participants who identified as Black or Pardo (mixed race) and among those living in households with lower per-capita income, cats, chickens, or reported rat sightings.

**Fig 4:**
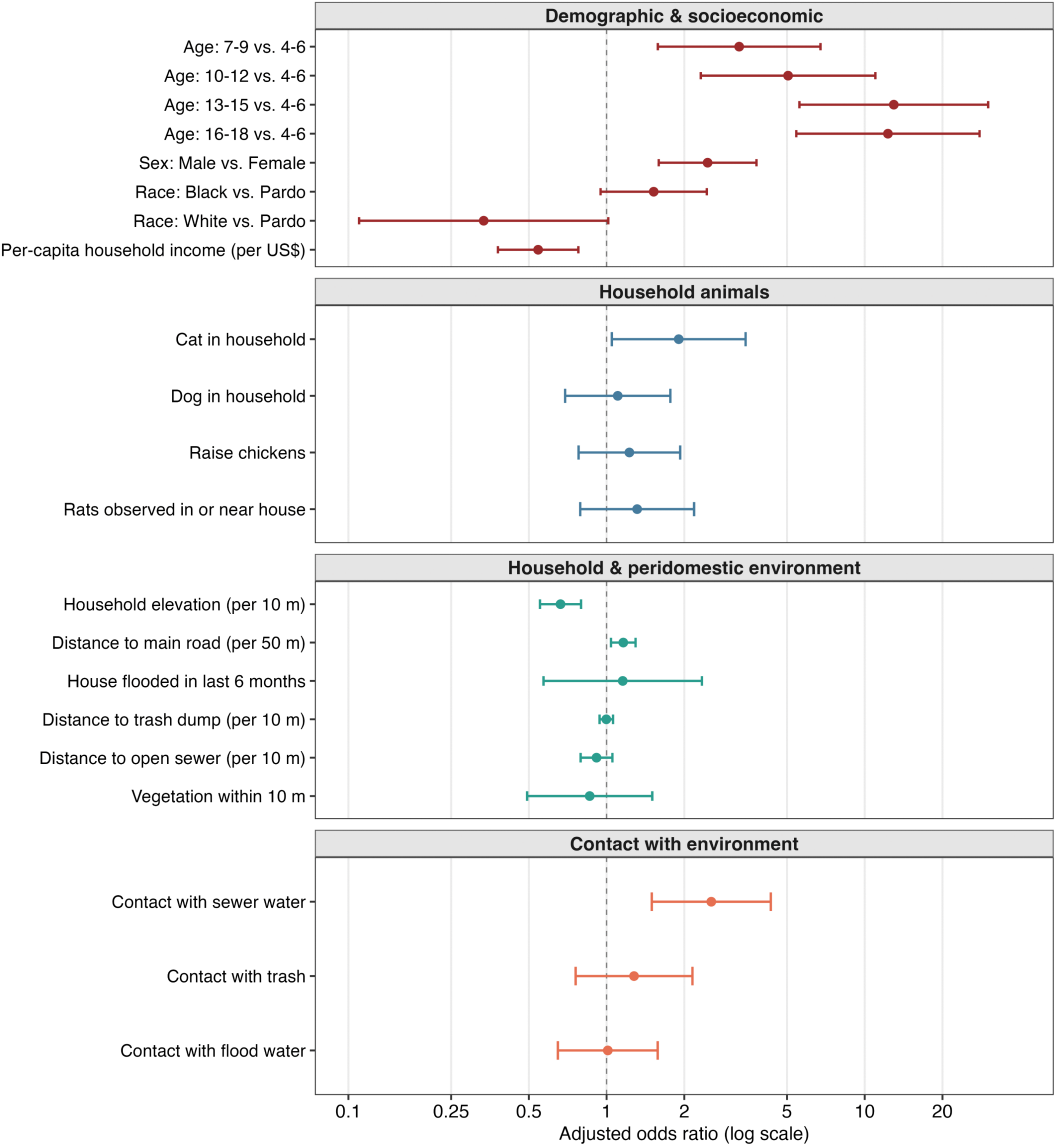
Forest plot of multivariable regression estimates of the total effect of each exposure on *T. gondii* seropositivity. Effect estimates and 95% confidence intervals are provided in S2 Table.

Estimates of the total effect of each exposure on seropositivity from the multivariable regression models, as defined by the assumed DAG, are shown in Fig 4 (see S2 Table for full set of model estimates).

Several demographic and socioeconomic variables were associated with seropositivity. Seropositivity increased with age, plateauing between the 13–15 and 16–18-year age groups (see Fig 4 and S2 Table). Male participants had more than twice the odds of seropositivity compared with female participants (OR 2.46; 95%CI 1.59, 3.81). Associations with race were imprecisely estimated and confidence intervals crossed the null, although point estimates suggested lower odds among White children and higher odds among Black children compared with Pardo children.

Indicators of social marginalisation were associated with seropositivity. Each US$1 increase in per-capita household income was associated with lower odds of seropositivity (OR 0.54; 95%CI 0.38, 0.78). Similarly, the odds of seropositivity increased by 16% (OR 1.16; 95%CI 1.04, 1.30) for every additional 50 metres of distance from the main road. Households located further from main roads are situated in areas characterised by greater poverty, limited infrastructure, mixed environmental conditions (including soil and open waterways), and reduced access to public services.

The seroprevalence among households with a cat (60.9%; 81/133) was higher than those without a cat (46.4%; 276/595) and participants living in households with a cat had higher odds of seropositivity (OR 1.93; 95%CI 1.08, 3.44). However, the majority of seropositive individuals (77.3%) did not live in households with a cat.

Household and peridomestic environmental characteristics, and reported contact with the environment were also associated with seropositivity. Living at higher household elevation, a proxy for decreased flooding risk and less environmentally degraded areas, was associated with lower odds of seropositivity, with each 10m increase in elevation corresponding to an OR of 0.66 (95%CI 0.55, 0.80). Participants reporting contact with sewer water near the household in the previous six months had 2.54 (95% CI 1.50, 4.33) times the odds of seropositivity compared with those reporting no such contact.

Model-based marginal predictions suggested higher seroprevalence among participants living in households with cats than those without cats (58.8% vs 45.8%), and among participants reporting contact with sewer water compared with those reporting no contact (61.0% vs 43.9%). These results are consistent with elevated exposure risk but the high exposure among unexposed groups indicating substantial risk beyond these specific exposures.

E-values for selected statistically significant associations (p < 0.05) ranged from 1.37 to 6.66 (S3 Table), indicating that an unmeasured confounder would need to be moderately to strongly associated with both the exposure and seropositivity (independently of measured covariates) to fully explain away these observed associations.

### Spatial modelling of heterogeneity in seroprevalence

**Fig 5:**
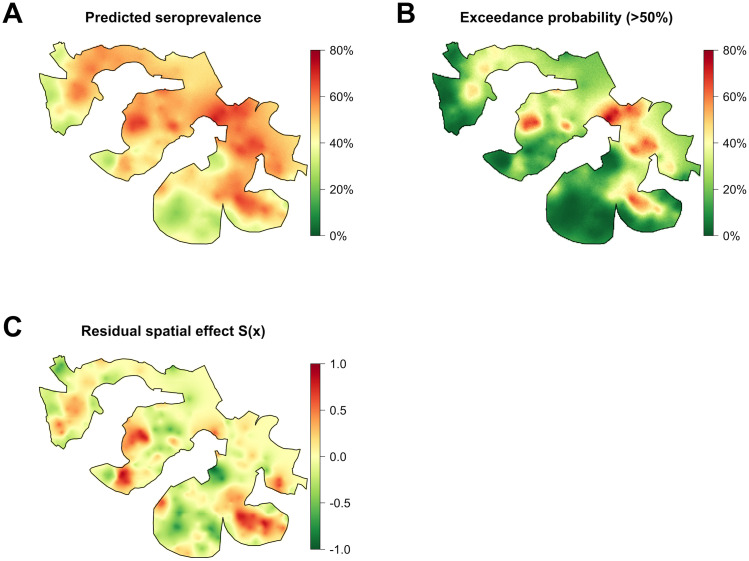
Spatial predictions from geostatistical models of *T. gondii* seroprevalence in Pau da Lima, Salvador, Brazil. **A)** Posterior mean seroprevalence predicted from the intercept-only model. **B)** Posterior exceedance probability that seroprevalence exceeds 50% from the intercept-only model, highlighting areas with a high probability of elevated seroprevalence. **C)** Posterior mean of the residual spatial random effect S(x) from the full model which included covariates from all four domains. The boundary outline was created by the authors for this study and does not contain copyrighted third-party material.

The intercept-only geostatistical model revealed substantial spatial heterogeneity in *T. gondii* seroprevalence across Pau da Lima, with areas of elevated predicted seroprevalence exceeding 60% (Fig 5A). Lower seroprevalence was predicted in the southernmost sections of the three valleys, in areas close to the main road. Exceedance probabilities (Fig 5B) indicated a high probability (>0.9) that seroprevalence exceeded 50% in several contiguous hotspots, especially in the central and northern sections of each valley.

The full geostatistical model included selected covariates (age, sex, household income, elevation, distance to road, cat ownership, and sewer water contact; see S4 Table for the AIC of the top five models) and attenuated, but did not eliminate, spatial clustering. The estimated spatial covariance parameters indicated residual short-range spatial structure in seroprevalence. The spatial correlation parameter, ϕ, decreased from 58.72m (95%CI 24.68, 139.71) in the intercept-only model to 29.95m (95%CI 15.48, 57.95) in the full model (see S5 and S6 Tables), corresponding to a spatial correlation range of approximately 90m (defined as the distance at which correlation declines to 5%) in the full model. This residual spatial structure was evident in the posterior mean of the spatial random effect S(x) (Fig 5C), which represents spatial variation in seroprevalence not explained by the measured covariates. Compared with the intercept-only model, the residual surface had fewer and smaller areas of elevated risk, but spatial heterogeneity persisted. These remaining areas of elevated risk are consistent with the influence of unmeasured environmental processes that vary over relatively short distances.

The distribution of residual variance further supported this interpretation. The nugget variance ($\tau^{\text{2}}$ = 1.48; 95%CI 0.62, 3.55) exceeded the spatially structured variance ($\sigma^{\text{2}}$ = 0.66; 95%CI 0.37, 1.18), indicating that approximately 31% of the residual variation was spatially structured, while the remaining 69% was attributable to unstructured variation captured by the nugget effect. This unstructured component represents heterogeneity operating at very fine spatial scales, consistent with household-level or immediate peridomestic processes not captured by the measured covariates. Taken together, these results indicate that residual heterogeneity in seroprevalence reflects contributions from both spatially structured processes operating over short distances and unstructured variation at very fine spatial scales.

## Discussion

In this study, we analysed trends in *T. gondii* seroprevalence among children and adolescents living in a low-income urban community and found evidence of substantial exposure, with nearly half of participants showing serological evidence of prior infection and moderate estimated annual forces of infection. Male sex, lower household income, cat ownership, residence further from the main road or at lower elevations, and reported contact with sewer water were all associated with seropositivity. Notably, however, most seropositive participants did not live in households with cats. Geostatistical analyses revealed marked spatial heterogeneity in seroprevalence over small spatial scales, with clustered hotspots exceeding 60% predicted prevalence. Adjustment for measured individual, household, and environmental factors attenuated but did not eliminate spatial clustering, indicating the presence of unmeasured environmental processes operating beyond individual households. Together, these findings suggest that *T. gondii* exposure represents a substantial health risk in marginalised urban communities and are consistent with an important role for social marginalisation and environmental degradation in shaping transmission through environmental and peridomestic exposure pathways.

Our findings suggest a significant risk of congenital toxoplasmosis in urban informal settlements with similar demographic and environmental features to those of our study site. Almost half of female participants remained unexposed upon reaching childbearing age, indicating a substantial susceptible population entering reproductive age. This risk is compounded by the relatively high force of infection, with an estimated 5% of susceptible females in our study population becoming infected annually. A sensitivity analysis found no evidence that the force of infection differed between ages 4–12 and 13–18 years, supporting the interpretation that exposure continues through adolescence and may be independent of age-specific behaviours. Primary infection during pregnancy can result in severe congenital disease, including long-term impairments in vision, behaviour, and cognitive function [9,10], highlighting the public health importance of understanding exposure pathways in these settings. However, because our data were restricted to children and adolescents, the estimated force of infection may not generalise to adult women of childbearing age, whose exposure patterns may differ due to behavioural changes, domestic roles, mobility, pregnancy-related factors, or depletion of the most highly exposed subgroups. More generally, the high exposure rates observed across childhood are also concerning given growing evidence linking latent infection to neuropsychiatric and behavioural outcomes [13].

Several findings were consistent with *T. gondii* exposure occurring through environmental pathways operating both within and beyond the household environment. Household cat ownership was associated with seropositivity, suggesting that intradomiciliary contamination from domestic cats may contribute to exposure in some cases. However, given that 77.3% of seropositive participants did not reside in households with cats, cat ownership alone cannot account for the observed burden of infection. Model-based marginal predictions indicated higher seroprevalence among participants living in households with cats compared with those without (approximately 59% versus 45%) but also demonstrated substantial exposure among children without direct household cat contact. These findings suggest that while domestic hygiene may be relevant in some households, exposure frequently occurs through broader contact with contaminated environments rather than through direct interaction with cats alone. This is consistent with observations in the study area that some cats are kept indoors, while others roam freely and defecate in the community. This interpretation is supported by associations with environmental characteristics indicative of wider exposure opportunities, including residence at lower elevations – where severe flooding is common [25] - and reported contact with sewer water; these factors likely increase contact with oocyst-contaminated soil or water in and around the household, particularly in environmentally degraded areas with poor drainage and sanitation infrastructure. Further evidence for environmental transmission is provided by a recent study of *T. gondii* exposure among animals in the same study area, which reported seroprevalence of 22.3% in cats and 66.7% in chickens. Chickens are infected almost exclusively by ingesting environmental oocysts while foraging and pecking at soil, so they can act as sentinels for local oocyst contamination. Their high seroprevalence therefore supports the hypothesis that transmission in this setting occurs through oocysts dispersed in the environment [18].

Spatial modelling provided further support for the role of environmentally mediated exposure beyond individual households. *T. gondii* seroprevalence exhibited clear clustering at sub-neighbourhood scales, and although adjustment for measured individual-, household-, and environmental-level covariates attenuated spatial clustering, residual spatial dependence persisted. This spatial pattern is more consistent with exposure to environmentally distributed oocysts than with food-borne exposure via tissue cysts, which would not be expected to show spatial structure at these spatial scales. The estimated spatial correlation range of approximately 90m indicated that shared exposure processes operate over distances extending beyond single households. This is consistent with an individual’s infection risk being affected by local environmental contamination caused by hydrology, sanitation, landscape features and the presence of cats in the area surrounding their household. The 90m range plausibly corresponds to the scale of local drainage catchments and possibly the home range of domestic cats in the area. At the same time, a substantial proportion of unexplained variation was unstructured, suggesting additional heterogeneity at the household or immediate peridomestic level. Together, these spatial patterns indicate that *T. gondii* exposure in this setting likely reflects a combination of shared environmental processes operating across small areas and household-level or peridomestic factors, including unmeasured behaviours and micro-environmental conditions that were not captured by available data.

The peridomestic environment in informal settlements can act as a persistent environmental reservoir that supports the survival of *T. gondii* oocysts, increasing residents’ exposure to contaminated soil and water. In Pau da Lima, the steep valley topography and open sewerage systems concentrate floodwater, sediment, and waste in low-lying areas, making household elevation and sewer contact effective proxies for hydrological processes that facilitate oocyst persistence and transport [38]. These conditions may promote spillover from households with cats to the surrounding environment, amplifying exposure risk beyond individual households. Similar hydrology-driven dispersal mechanisms have been documented for other environmentally persistent pathogens in this community, including *Leptospira* [25,39,40], and are supported by studies in Chile, France, and Brazil linking flooding, soil contamination, and environmental exposure to *T. gondii* seropositivity and congenital toxoplasmosis [21,22,41–43].

Addressing these environmental deficiencies through improved sanitation, drainage, and flood mitigation infrastructure may consequently be important for limiting environmental contamination and reducing exposure risk. As climate change is expected to increase the frequency and intensity of flooding events in tropical urban settings, understanding how hydrology, hygiene, and environmental reservoirs interact may be increasingly critical for preventing toxoplasmosis in climate-vulnerable informal settlements.

Social marginalisation appeared to play an important role in shaping exposure risk within the study community. Increased risk of seropositivity was associated with living in households with lower per-capita income, at lower elevations, and further from the main road. These areas are characterised by poorer infrastructure and reduced access to services. In the steep valleys common to informal settlements in urban Brazil, distance from main roads reflects both physical and social marginalisation. Although associations between socioeconomic vulnerability and *T. gondii* exposure have been reported previously [21,44–47], it is notable that substantial risk gradients were observed over small spatial distances within a low-income community. Socioeconomic constraints may limit where families can reside, confining them to environments with frequent contact with contaminated soil and water, high densities of intermediate rodent hosts, and limited infrastructure to support effective hygiene practices [44]. Race may also be relevant to *T. gondii* exposure in this setting, although adjusted associations were imprecise. Larger studies should examine whether race shapes infection risk through structural differences in household, socioeconomic, and environmental conditions.

The association between male sex and increased *T. gondii* exposure risk has not been widely reported in other studies globally. In Pau da Lima, males have been shown to experience higher risk of leptospirosis, likely reflecting differences in mobility patterns, risk perception, and behaviours [48,49]. Although such gender differences are more commonly documented in adults, our findings suggest that similar behavioural factors may also influence exposure among children and adolescents, particularly through differential contact with contaminated environments and hygiene practices. These behaviours are likely to vary more by sex than dietary habits, which may help explain the observed sex difference in seroprevalence [44].

This study contributes to a limited body of research on *T. gondii* exposure in urban informal settlements, particularly among children and adolescents. Although the data were collected in 2003, the social, environmental and infrastructural conditions characterising the study community continue to be prevalent across informal settlements in Salvador and globally. The findings are therefore likely to be relevant to other marginalised urban contexts with similar social, environmental and sanitary conditions. This is supported by seroprevalence estimates from comparable communities internationally, which are similar to those observed in this study [22,45,47,50,51]. Together, these findings provide a valuable reference point for understanding contemporary transmission dynamics and for contextualising more recent studies conducted in comparable environments.

Several limitations should be considered. The cross-sectional design and use of seropositivity (i.e., as a marker of historical exposure) as an outcome may introduce temporality bias, as exposure may have occurred prior to measurement of some explanatory variables or at previous residences. Some variables were self-reported or proxied, and residual confounding - such as dietary exposures or other unmeasured behaviours - may remain. We did not directly measure environmental contamination with oocysts and instead relied on environmental and behavioural proxies to infer transmission pathways.

Household geolocation error may have introduced exposure misclassification for distance-based environmental covariates, such as distance to sewer, where assigned values may be sensitive to small positional errors. As any such error is unlikely to be associated with seropositivity, it would likely be non-differential and bias associations towards the null, although it may have contributed to the nugget effect in the geostatistical model. The serocatalytic model also assumed a constant force of infection over calendar time. As Pau da Lima underwent urbanisation and infrastructure change before the 2003 survey, age associations may partly reflect historical living conditions rather than only the environment measured at sampling. Consequently, further longitudinal research, particularly among women of childbearing age, is needed to characterise current age-specific exposure dynamics and quantify the risk of primary infection during pregnancy in high-risk urban populations.

This study underscores the neglected status of toxoplasmosis in urban informal settings and highlights the need for research and public health strategies that explicitly address environmental transmission pathways. The high estimated force of infection and substantial proportion of women remaining susceptible into reproductive age indicate a significant risk of primary infection during pregnancy, underscoring the importance of improved surveillance and prevention strategies for congenital toxoplasmosis in these settings.

Our findings suggest that interventions limited to households with domestic cats are unlikely to be sufficient. Identified associations with indicators of environmental exposure, and fine-scale spatial clustering point to the need for broader approaches that reduce environmental contamination beyond an individual household (e.g., improved sanitation, drainage, and flood mitigation). At the household and peridomestic level, measures that reduce contact with contaminated sewer and flood water, alongside improved hygiene practices and waste management may help reduce exposure. Future work would benefit from a One Health framework that integrates human, animal, and environmental data to better delineate transmission routes and identify intervention points. Effective prevention in these settings will require strategies that address social marginalisation and environmental degradation at both household and community levels, rather than focusing solely on individual behaviours or domestic animal ownership [52].

## Supporting information

S1 Fig STROBE (Strengthening the Reporting of Observational Studies in Epidemiology) flowchart showing the recruitment of study participants. (DOCX)

S1 Table Domains, variables, and rationale for explanatory variables used in the analysis. Variables were grouped into four domains that capture both the potential sources of *T. gondii* oocysts and the places where people are likely exposed to them.
(DOCX)

S2 Fig Generalized Additive Model (GAM) partial dependence plots for the exploratory analysis to identify the functional form of continuous explanatory variables against the log-odds of seropositivity for: A. age; B. distance to the main road; C. distance to nearest trash dump; D. distance to nearest waste sewer; E. household elevation in meters; F. per-capita household income. (DOCX)

S3 Fig Full Directed Acyclic Graph (DAG) for *T. gondii* serostatus in children and adolescents with direction of causality indicated by arrows (available online at https://dagitty.net/m5z6UTbAP). (DOCX)

S1 Appendix Geostatistical modelling framework. (DOCX)

S2 Table Multivariable regression estimates of the total effect of each exposure on *T. gondii* seropositivity, informed by causal diagrams and grouped by exposure domains (as outlined in S1 Table). (DOCX)

S3 Table E-values for selected multivariable associations between exposures and *T. gondii* seropositivity. (DOCX)

S4 Table Model selection for the spatial analysis: Multivariable mixed-effects logistic regression selection table ordered by corrected Akaike Information Criteria (AICc) with the degrees of freedom (df) and difference in AICc relative to the top ranked model (delta). (DOCX)

S5 Table Intercept-only geostatistical model parameter estimates. (DOCX)

S6 Table Full geostatistical model parameter estimates. (DOCX)
