# Supplementary material for: Social marginalisation, environmental degradation and Toxoplasma gondii exposure in urban informal settlements in Brazil
Source: PLoS Negl Trop Dis. 2026 Jun 22;20(6):e0014453. doi: 10.1371/journal.pntd.0014453 (PMC13309048; doi:10.1371/journal.pntd.0014453)
Supplement: S1 Fig — (DOCX) [file pntd.0014453.s001.docx]

**S1 Fig**. STROBE (Strengthening the Reporting of Observational Studies in Epidemiology) flowchart showing the recruitment of study participants.
