# Supplementary material for: Social marginalisation, environmental degradation and Toxoplasma gondii exposure in urban informal settlements in Brazil
Source: PLoS Negl Trop Dis. 2026 Jun 22;20(6):e0014453. doi: 10.1371/journal.pntd.0014453 (PMC13309048; doi:10.1371/journal.pntd.0014453)
Supplement: S1 Table — Variables were grouped into four domains that capture both the potential sources of T. gondii oocysts and the places where people are likely exposed to them. (DOCX) [file pntd.0014453.s002.docx]

**S1 Table.** Domains, variables, and rationale for explanatory variables used in the analysis. Variables were grouped into four domains that capture both the potential sources of *T. gondii* oocysts and the places where people are likely exposed to them.

| **Domain** | **Variables** | **Rationale** |
| --- | --- | --- |
| **Demographic & socioeconomic** | Age, sex, race, household income, house ownership | Upstream determinants of exposure related to behaviour, social marginalisation, and household environment, which also act as confounders of associations between environmental pathways and serostatus. |
| **Household animals** | Cat in household, dog in household, raise chickens, observation of rats | **Source of oocysts:** shed by cats into the domestic and peridomestic environment; dogs, chickens, and rats as indicators of domestic and peridomestic sanitary conditions and potential amplification pathways (dogs as mechanical carriers; rats and chickens via predation increasing cat infection risk).  **Exposure site:** inside the house or immediate yard/peridomestic area |
| **Household & peridomestic environment** | House flooded in last 6 months, elevation of household, distance to trash dump, distance to open sewer, distance to main road, vegetation within 10m | **Source of oocysts:** shed by cats into the community environment, transported by flood water and sewage; trash dumps represent areas with high rat densities and increased risk of cat infection.  **Exposure site:** household, peridomestic and areas near the household; elevation represents risk of flooding; household flooding represents intrusion of contaminated external water into homes; distance to main road represents the lack of basic urban services; vegetation represents non-paved areas where soil, shade and moisture increase oocyst survival. |
| **Contact with environment** | Contact with trash, flood water or sewer water near household in the last 6 months | **Source of oocysts:** shed by cats into the community environment, transported by flood water and sewage.  **Exposure site:** peridomestic and areas near the household; exposure assumed to occur via direct contact with contaminated solid waste, flood water, or sewer water near to the household. |
|  | | |

**Demographic and socioeconomic characteristics**

Age, sex, race, household income, and house ownership were included as upstream determinants of exposure and susceptibility. These variables capture structural and social dimensions of marginalisation that shape behaviours, living conditions, and access to infrastructure, and may also act as confounders of associations between environmental exposure pathways and serostatus.

**Household animals**

The household animals domain represents potential sources and amplifiers of oocyst contamination within the domestic and immediate peridomestic environment. Cats were included as the definitive host and primary source of oocyst shedding. Dogs, chickens, and rats were considered indicators of domestic and peridomestic sanitary conditions and potential amplification pathways: dogs may act as mechanical carriers of oocysts, while chickens and rats can serve as intermediate hosts, increasing the risk of cat infection through predation.

**Household and peridomestic environment**

This domain captures environmental conditions in and immediately surrounding the household that may influence exposure risk. Variables include recent household flooding, household elevation, distance to trash dumps, distance to open sewers, distance to main roads, and nearby vegetation. These factors represent pathways through which oocysts shed into the wider environment may be transported via flooding, sewage, or runoff and persist in shaded, moist environments. Flooding was classified in this domain because exposure arises from external water intruding into the household, reflecting contamination originating in surrounding public spaces. Distance to main roads was included as a proxy for social and environmental marginalisation, reflecting reduced infrastructure, services, and waste management in areas further from main roads.

**Contact with the environment**

The contact-with-environment domain captures behaviours that bring children into direct contact with potentially oocyst-contaminated environments near the household, including contact with trash, flood water, or sewer water in the previous six months. These variables represent direct exposure opportunities through interaction with contaminated soil, water, or waste in peridomestic or nearby public spaces. Contact with sewer water captured environmental, rather than occupational, exposure to the open sewer channels running through the community, which residents may encounter during daily movement through the neighbourhood.
