# Supplementary material for: Social marginalisation, environmental degradation and Toxoplasma gondii exposure in urban informal settlements in Brazil
Source: PLoS Negl Trop Dis. 2026 Jun 22;20(6):e0014453. doi: 10.1371/journal.pntd.0014453 (PMC13309048; doi:10.1371/journal.pntd.0014453)
Supplement: S2 Fig — (DOCX) [file pntd.0014453.s003.docx]

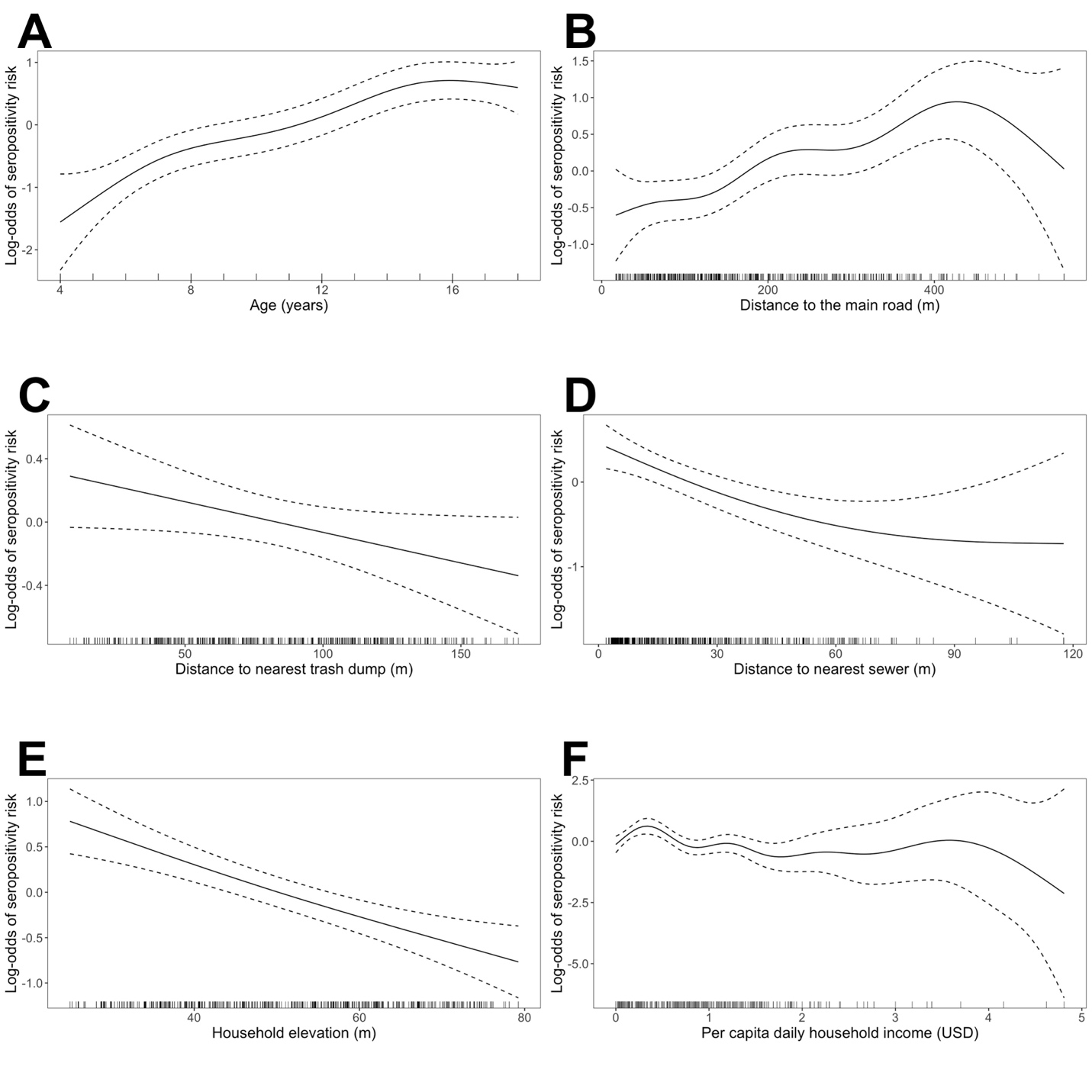


**S2 Fig**. Generalized Additive Model (GAM) partial dependence plots for the exploratory analysis to identify the functional form of continuous explanatory variables against the log-odds of risk of seropositivity for: A. age; B. distance to the main road; C. distance to nearest trash dump; D. distance to nearest waste sewer; E. household elevation in meters; F. per-capita household income.
