# Supplementary material for: Social marginalisation, environmental degradation and Toxoplasma gondii exposure in urban informal settlements in Brazil
Source: PLoS Negl Trop Dis. 2026 Jun 22;20(6):e0014453. doi: 10.1371/journal.pntd.0014453 (PMC13309048; doi:10.1371/journal.pntd.0014453)
Supplement: S3 Fig — (DOCX) [file pntd.0014453.s004.docx]

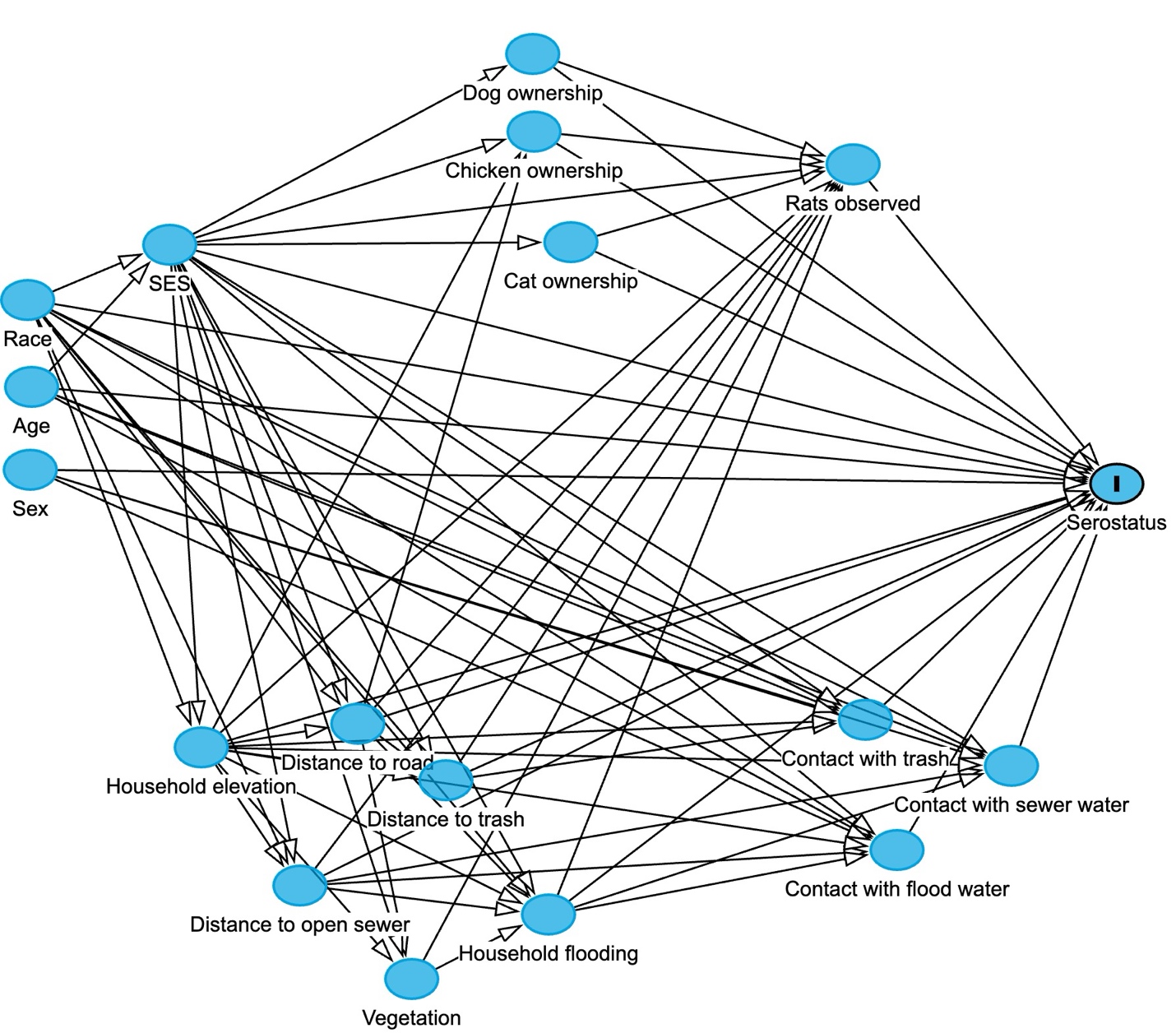


**S3 Fig.** Full Directed Acyclic Graph (DAG) for *T. gondii* serostatus in children and adolescents (available online at <https://dagitty.net/m5z6UTbAP>) with direction of causality indicated by arrows.
