# Supplementary material for: Social marginalisation, environmental degradation and Toxoplasma gondii exposure in urban informal settlements in Brazil
Source: PLoS Negl Trop Dis. 2026 Jun 22;20(6):e0014453. doi: 10.1371/journal.pntd.0014453 (PMC13309048; doi:10.1371/journal.pntd.0014453)
Supplement: S1 Appendix — (DOCX) [file pntd.0014453.s005.docx]

## **S1 Appendix. Geostatistical modelling framework**

**1. The geostatistical model**

We model the binary human seropositivity outcome as a Bernoulli variable with the probability, $p_{j}\left( x_{i} \right)$, that individual $j$ at location $x_{i}$ is seropositive. Our model for the variation in $p_{j}\left( x_{i} \right),$throughout the region of interest is that

$logit(p_{j}\left( x_{i} \right))=d\left( x_{i} \right)^{\top}\beta+ {e_{ij}}^{\top}\gamma+S\left( x_{i} \right)+Z_{i}$ Eqn (1)

In Equation (1), $d\left( x_{i} \right)$ is a vector of household-level covariates associated with regression coefficients β and $e_{ij}'$ is a vector of individual-level covariates associated with regression coefficients $\gamma$. This component of the model accounts for variation in prevalence that can be explained by measured characteristics of the individual at location $x$. The terms $S\left( x \right)$ and $Z$ account for any remaining variation that cannot be explained by measured characteristics of $x$. The term $S\left( x \right)$ is a spatially correlated Gaussian process with mean zero and covariance structure $Cov\left( S\left( x \right),S\left( x' \right) \right)=\sigma^{2}\rho\left( u;\phi\right),$ where $u=\|x-x'\|$ is the Euclidean distance between $x$ and $x^{'},$ $\sigma^{2}$ is the variance and $\rho\left( u;\phi\right)=exp(-u/\phi)$ is the correlation between $S\left( x \right)$ and $S\left( x' \right)$. The term $Z_{i}$ in Equation (1) is a Normally distributed random variable with zero mean and variance $\tau^{2}$ that varies independently between household locations; it is also known as the nugget effect.

**2. Parameter estimation**

We carry out parameter estimation using Monte Carlo Maximum Likelihood (MCML), implemented in PrevMap, an R package for analysing prevalence data, freely available from the Comprehensive R Archive Network ([*www.r-project.org*](http://www.r-project.org)).

We denote by $x_{1},\ldots,x_{n}$ the set of sampled locations. Let $\eta_{i}=log\left( p_{j}\left( x_{i} \right)/(1-p_{j}\left( x_{i} \right) \right)$. The joint conditional density of $Y=Y_{1},\ldots,Y_{n}$is $f\left( y|\eta\right)=\prod_{i=1}^{n} f\left( y_{i}|\eta_{i} \right)$.

The likelihood function for the set of model parameters $\psi$ is obtained by integrating out the random components$S\left( x_{i} \right)$and$Z_{i}$ from $\eta_{i}$, hence

$L\left( \psi\right)=\int_{R^{n}} f\left( y|\eta\right) f\left( \eta;\psi\right)d\eta$ Eqn (2)

where$f\left( \eta;\psi\right)$is a multivariate Normal density.

To approximate the integral in Equation 2 we use a Markov Chain Monte Carlo (MCMC) algorithm to generate a sample $\eta_{\left( 1 \right)},...,\eta_{\left( N \right)}$ from the conditional distribution of $\eta$ given $y$ and approximate the likelihood as

$$L\left( \psi\right)\propto L_{N}\left( \psi\right)=\frac{1}{N}\frac{\sum_{j=1}^{N} f\left( \eta_{\left( j \right)};\psi\right)}{f\left( \eta_{\left( j \right)};\psi_{0} \right)},$$

Where $\psi_{0}$ is our best guess for the initial parameter values.

**3. Prediction**

Here, we use plug-in prediction, meaning that we use the Monte Carlo maximum likelihood parameter estimate $\psi^$ in place of the unknown $\psi$.

Our goal is to predict prevalence throughout the region of interest, $A$. We approximate this by a regular grid of points $x_{n+1},...,x_{n+q}$ that cover $A$. Our predictive target is the set of values

$\eta_{n+i}=d\left( x_{n+i} \right)'\beta+S\left( x_{n+i} \right)$ Eqn (3)

Note that Equation 3 excludes the term $Z$ in Equation 1, which relates to characteristics of the sampled individuals at a location rather than of the location itself.

The *predictive distribution* of $\eta^{*}=\left( \eta_{n+1},...,\eta_{n+q} \right)$ is its conditional distribution given $y$,

$$f\left( \eta^{*}|y \right)=\int_{R^{n}} f\left( \eta^{*}|\eta\right)f\left( \eta|y \right)d\eta,$$

where we have used the fact that $\eta^{*}$ and $y$ are conditionally independent given $\eta$. It follows that to generate a sample from the predictive distribution of $\eta^{*}$ we first sample from $f\left( \eta|y \right)$ and then from$f\left( \eta^{*}|\eta\right)$.
