# Supplementary material for: Social marginalisation, environmental degradation and Toxoplasma gondii exposure in urban informal settlements in Brazil
Source: PLoS Negl Trop Dis. 2026 Jun 22;20(6):e0014453. doi: 10.1371/journal.pntd.0014453 (PMC13309048; doi:10.1371/journal.pntd.0014453)
Supplement: S2 Table — (DOCX) [file pntd.0014453.s006.docx]

**S2 Table.** Multivariable regression estimates of the total effect of each exposure on *T. gondii* seropositivity, informed by causal diagrams and grouped by exposure domains (as outlined in S1 Table).

| **Variables** | **aOR (95% CI)** |
| --- | --- |
| **Demographic & socioeconomic** | |
| Age (years)^1^ |  |
| 4-6 | REF |
| 7-9 | 3.26 (1.58, 6.74) |
| 10-12 | 5.05 (2.32, 11.00) |
| 13-15 | 12.96 (5.58, 30.08) |
| 16-18 | 12.30 (5.43, 27.85) |
| Sex^2^ |  |
| Female | REF |
| Male | 2.46 (1.59, 3.81) |
| Race^3^ |  |
| Pardo (mixed) | REF |
| Black | 1.52 (0.95, 2.45) |
| White | 0.33 (0.11, 1.02) |
| Other | 1.00 (0.02, 49.34) |
| Per-capita daily household income in US$^2^ | 0.54 (0.38, 0.78) |
| **Household animals** | |
| Cat in household^4^ | 1.93 (1.08, 3.44) |
| Dog in household^4^ | 1.06 (0.67, 1.67) |
| Raise chickens^5^ | 1.28 (0.82, 1.99) |
| Observation of rats in or near house^6^ | 1.31 (0.79, 2.18) |
| **Household & peridomestic environment** |  |
| Household elevation (per 10m)^4^ | 0.66 (0.55, 0.80) |
| Distance to the main road (per 50m)^7^ | 1.16 (1.04, 1.3) |
| House flooded in last 6 months^8^ | 1.04 (0.52, 2.09) |
| Distance to nearest trash dump (per 10m)^5^ | 1.00 (0.94, 1.06) |
| Distance to nearest open sewer (per 10m)^6^ | 0.91 (0.79, 1.05) |
| Vegetation within 10m of the house^5^ | 0.85 (0.49, 1.47) |
| **Contact with environment** |  |
| Contact with sewer water^9^ | 2.54 (1.50, 4.33) |
| Contact with trash^10^ | 1.28 (0.76, 2.15) |
| Contact with flood water^9^ | 1.01 (0.65, 1.58) |
| *Adjusted Odds Ratio (aOR)  Adjustment sets: ^1^sex, race; ^2^age, race; ^3^sex, age; ^4^age, race, income; ^5^age, race, income, elevation, distance to road; ^6^age, race, income, elevation, distance to road, distance to sewer, vegetation, distance to trash pile, cat, dog, chicken; ^7^age, race, income, elevation; ^8^age, race, income, elevation, distance to road, distance to sewer, vegetation; ^9^age, race, sex, income, elevation, distance to road, distance to sewer, house flooded, vegetation; ^10^age, race, sex, income, elevation, distance to road, distance to trash pile; | |
