# Supplementary material for: Social marginalisation, environmental degradation and Toxoplasma gondii exposure in urban informal settlements in Brazil
Source: PLoS Negl Trop Dis. 2026 Jun 22;20(6):e0014453. doi: 10.1371/journal.pntd.0014453 (PMC13309048; doi:10.1371/journal.pntd.0014453)
Supplement: S3 Table — (DOCX) [file pntd.0014453.s007.docx]

**S3 Table.** E-values for selected multivariable associations between exposures and *T. gondii* seropositivity.

| **Variables** | **aOR (95% CI)** | **E-value** |
| --- | --- | --- |
| **Demographic & socioeconomic** | |  |
| Age (years) |  |  |
| 4-6 | REF |  |
| 7-9 | 3.26 (1.58, 6.74) | 3.01 |
| 10-12 | 5.05 (2.32, 11.00) | 3.92 |
| 13-15 | 12.96 (5.58, 30.08) | 6.66 |
| 16-18 | 12.30 (5.43, 27.85) | 6.47 |
| Sex |  |  |
| Female | REF |  |
| Male | 2.46 (1.59, 3.81) | 2.51 |
| Per-capita daily household income in US$^1^ | 0.54 (0.38, 0.78) | 2.05 |
| **Household animals** | |  |
| Cat in household | 1.93 (1.08, 3.44) | 2.12 |
| **Household & peridomestic environment** |  |  |
| Household elevation (per 10m) | 0.66 (0.55, 0.80) | 1.76 |
| Distance to the main road (per 50m) | 1.16 (1.04, 1.3) | 1.37 |
| **Contact with environment** |  |  |
| Contact with sewer water | 2.54 (1.50, 4.33) | 2.57 |
| ^1^For odds ratios below 1, the E-value corresponds to the minimum strength of association on the risk ratio scale that an unmeasured confounder would need to have with both income and seropositivity to explain away the observed protective association. | |  |
