# Supplementary material for: Social marginalisation, environmental degradation and Toxoplasma gondii exposure in urban informal settlements in Brazil
Source: PLoS Negl Trop Dis. 2026 Jun 22;20(6):e0014453. doi: 10.1371/journal.pntd.0014453 (PMC13309048; doi:10.1371/journal.pntd.0014453)
Supplement: S4 Table — (DOCX) [file pntd.0014453.s008.docx]

**S4 Table.** Model selection for the spatial analysis: Multivariable mixed-effects logistic regression selection table ordered by corrected Akaike Information Criteria (AICc) with the degrees of freedom (df) and difference in AICc relative to the top ranked model (delta).

| **Model ranking** | **Variables** | **df** | **AICc** | **Delta** |
| --- | --- | --- | --- | --- |
| 1 | Age, sex, income, cat, elevation, distance to main road, contact with sewer water | 12 | 858.97 | 0 |
| 2 | Age, sex, income, elevation, distance to main road, contact with sewer water | 11 | 859.46 | 0.49 |
| 3 | Age, sex, income, cat, distance to main road, contact with sewer water | 11 | 861.08 | 2.12 |
| 4 | Age, sex, income, distance to main road, contact with sewer water | 10 | 862.34 | 3.37 |
| 5 | Age, sex, cat, elevation, distance to main road, contact with sewer water | 11 | 862.52 | 3.56 |
