# Supplementary material for: Social marginalisation, environmental degradation and Toxoplasma gondii exposure in urban informal settlements in Brazil
Source: PLoS Negl Trop Dis. 2026 Jun 22;20(6):e0014453. doi: 10.1371/journal.pntd.0014453 (PMC13309048; doi:10.1371/journal.pntd.0014453)
Supplement: S5 Table — (DOCX) [file pntd.0014453.s009.docx]

**S5 Table.** Intercept-only geostatistical model parameter estimates

| **Parameter** |  |
| --- | --- |
| **Spatial parameters** | **Estimate (95% CI)** |
| ϕ (scale of spatial correlation, metres) | 58.72 (24.68, 139.71) |
| $\sigma^{2}$ (variance of the Gaussian process) | 0.76 (0.46, 1.25) |
| $\tau^{2}$ (variance of the nugget effect) | 0.75 (0.44, 2.17) |
