# Supplementary material for: Social marginalisation, environmental degradation and Toxoplasma gondii exposure in urban informal settlements in Brazil
Source: PLoS Negl Trop Dis. 2026 Jun 22;20(6):e0014453. doi: 10.1371/journal.pntd.0014453 (PMC13309048; doi:10.1371/journal.pntd.0014453)
Supplement: S6 Table — (DOCX) [file pntd.0014453.s010.docx]

**S6 Table.** Full geostatistical model parameter estimates

| **Parameter** |  |
| --- | --- |
| **Variables** | **OR (95% CI)** |
| Age (years) |  |
| 10-12 | REF |
| 4-6 | 0.15 (0.08, 0.30) |
| 7-9 | 0.5 (0.29, 0.87) |
| 13-15 | 2.11 (1.17, 3.80) |
| 16-18 | 2.70 (1.54, 4.72) |
| Sex |  |
| Female | REF |
| Male | 2.33 (1.51, 3.53) |
| Per capita daily household income in US$ | 0.70 (0.53, 0.93) |
| Elevation of household (per 10m) | 0.86 (0.74, 1.00) |
| Distance to the main road (per 10m) | 1.04 (1.02, 1.06) |
| Cat in household | 1.53 (0.93, 2.53) |
| Contact with sewer water | 2.21 (1.43, 3.41) |
| **Spatial parameters** | **Estimate (95% CI)** |
| ϕ (scale of spatial correlation, metres) | 29.95 (15.48, 57.95) |
| $\sigma^{2}$ (variance of the Gaussian process) | 0.66 (0.37, 1.18) |
| $\tau^{2}$ (variance of the nugget effect) | 1.48 (0.62, 3.55) |
